# Draft Genome Sequence of *Bacillus luciferensis* Isolated from Soil

**DOI:** 10.1128/genomeA.01140-16

**Published:** 2016-10-20

**Authors:** Loni Townsley, Lews Caro, Hemant Kelkar, Elizabeth A. Shank

**Affiliations:** a Department of Biology, University of North Carolina, Chapel Hill, North Carolina, USA; b Department of Genetics, University of North Carolina, Chapel Hill, North Carolina, USA; c Department of Microbiology and Immunology, University of North Carolina, Chapel Hill, North Carolina, USA; d Curriculum of Genetics and Molecular Biology, University of North Carolina, Chapel Hill, North Carolina, USA

## Abstract

*Bacillus luciferensis* is a Gram-positive, facultatively anaerobic, motile rod. Here, we report the first draft genome sequence, to our knowledge, of a *B. luciferensis* strain (CH01), which will provide useful information for *Bacillus* and soil bacteria research.

## GENOME ANNOUNCEMENT

*Bacillus* species are Gram-positive endospore-forming bacteria noted for their extensive range of physiological abilities and wide distribution in the natural environment, including temperate, acidic, neutral, and alkaline soils; fresh and marine waters; and clinical specimens (1). In soil, *Bacillus* spp. participate in interspecies interactions that influence important cellular processes in other bacteria in the soil community (2–6). They can also promote growth and elicit induced systemic resistance (ISR) in plants (7, 8). Thus, bacteria from the *Bacillus* genus are important for microbial ecology and agricultural applications.

*Bacillus luciferensis* was originally identified in the soil from Lucifer Hill, a volcano on Candlemas Island, South Sandwich Islands (9). We isolated *B. luciferensis* strain CH01 from soil samples obtained in Chapel Hill, NC, USA. Genomic DNA was extracted using the Genomic DNA purification kit (Promega, Madison, WI, USA). Library preparation and genome sequencing were performed at the University of North Carolina (UNC) School of Medicine, High-Throughput Sequencing Facility (HTSF). A total of 3,202,628 paired-end reads were generated using the Illumina MiSeq platform. Initial quality control for sequence data was performed using FastQC (10). Adaptor sequence removal, trimming, error correction, and *de novo* assembly were performed using the A5-miseq pipeline (11) and analyzed with QUAST (12). The assembly produced 38 contigs, with a total length of 4,899,652 bp, maximum length of 506,555 bp, *N*_50_ of 299,167 bp, and a G+C content of 32.96%. *De novo* annotation was performed using the NCBI Prokaryotic Genome Annotation Pipeline (http://www.ncbi.nlm.nih.gov/genome/annotation_prok). One intact prophage was identified using PHAST (13). Potential secondary metabolites identified using antiSMASH (14) included a type III polyketide synthase (PKS), a microcin, a siderophore, a bacteriocin, and two terpene clusters. This genome can be used for comparative analysis of *Bacillus* species to broaden our understanding of the diversity of the genus, and it will enable further study of the genetic and functional characteristics of *B. luciferensis.*

### Accession number(s).

The annotated genome sequence has been deposited in GenBank under the accession number MDKC00000000.
